# Supplementary material for: Comparative Study of Potential Habitats for Simulium qinghaiense (Diptera: Simuliidae) in the Huangshui River Basin, Qinghai–Tibet Plateau: An Analysis Using Four Ecological Niche Models and Optimized Approaches
Source: Insects. 2024 Jan 23;15(2):81. doi: 10.3390/insects15020081 (PMC10889266; doi:10.3390/insects15020081)
Supplement: Supplementary file 1 [file insects-15-00081-s001.zip › insects-2817798-supplementary.pdf]

Comparative Study of Potential Habitats for *Simulium qinghaiense* (Diptera: Simuliidae) in the Huangshui River Basin, Qinghai–Tibet Plateau: An Analysis Using Four Ecological Niche Models and Optimized Approaches

Yunxiang Liu [1,2,†], Chuanji Li [1,2,†] and Hainan Shao [1,*]

Supplementary Materials

Legends for Tables and Figures

Table S1. Field survey sampling site information

Province	Survey sites	Sample points	Longitude	Latitude	Altitude(m)	Sample size
Qinghai	Haiyan County (HYA)	6	100.8512	37.3012	3838	227
			100.7504	37.1507	3521	170
			100.8615	37.0446	3198	220
			100.994	36.9981	3089	174
			101.0978	36.8202	2978	175
			100.7872	37.0434	3285	164
	Huangyuan County (HY)	2	101.1427	36.6943	3051	212
			101.2842	36.7161	3110	224
	Huangzhong County (HZA)	4	101.3369	36.6183	3126	226
			101.4822	36.6475	2402	190
			101.3823	36.8197	2786	156
			101.618	36.5812	2447	212
	Xining City (XN)	1	101.7719	36.6343	2238	182
	Huzhu County (HZ)	2	101.9216	36.6074	2541	225
			102.137	36.5987	2677	219
	Pingan District (PA)	3	101.9073	36.5082	2704	158
			102.0295	36.4851	2404	181
			102.1606	36.4799	2152	182
	Ledu District (LD)	5	102.327	36.5389	2193	223
			102.4307	36.4629	2052	207
			102.5738	36.3527	2226	174
			102.4137	36.6115	2614	214
			102.7675	36.4598	2416	207

	Minhe County (MH)	3	102.745	36.3554	1905	110
			102.6473	36.2198	2391	103
			102.9349	36.2906	1715	310
	Qilian Mountions (QLM)	0	\	\	\	0
	Tianjun County (TJ)	0	\	\	\	0
	Gangcha County (GC)	0	\	\	\	0
	Qilian County (QL)	0	\	\	\	0
	Menyuan County (MY)	0	\	\	\	0
	Datong County (DT)	0	\	\	\	0
Gansu	Yongdeng County (YD)	1	103.2662	36.3289	1898	226
	Honggu District (HG)	2	103.3343	36.2299	1831	195
			103.2135	36.206051	1804	158
	Yongjing County (YJ)	1	103.0078	36.1724	2032	223
	Tianzhu County (TZ)	0	\	\	\	0

Table S2. Environment variable

Environmental variable	Variable description	Unit
bio1	Annual Mean Temperature	℃
bio2	Mean Diurnal Range	℃
bio3	Isothermality	\
bio4	Temperature Seasonality	\
bio5	Max Temperature of Warmest Month	℃
bio6	Min Temperature of Coldest Month	℃
bio7	Temperature Annual Range	℃
bio8	Mean Temperature of Wettest Quarter	℃
bio9	Mean Temperature of Driest Quarter	℃
bio10	Mean Temperature of Warmest Quarter	℃
bio11	Mean Temperature of Coldest Quarter	℃
bio12	Annual Precipitation	mm
bio13	Precipitation of Wettest Month	mm
bio14	Precipitation of Driest Month	mm
bio15	Precipitation Seasonality	\
bio16	Precipitation of Wettest Quarter	mm
bio17	Precipitation of Driest Quarter	mm
bio18	Precipitation of Warmest Quarter	mm
bio19	Precipitation of Coldest Quarter	mm
Alt	Altitude	m

Table S3. Selected environmental variables post-filtering

Environmental variable	Variable description	Unit
bio2	Mean Diurnal Range	℃

bio9	Mean Temperature of Driest Quarter	℃
bio12	Annual Precipitation	mm
bio14	Precipitation of Driest Month	mm
bio15	Precipitation Seasonality	\
bio18	Precipitation of Warmest Quarter	mm
bio19	Precipitation of Coldest Quarter	mm
Alt	Altitude	m

Table S4. Single-sample Kolmogorov–Sminov test

		AUC	Kappa
N		40	40
Normal parameters a, b	Average	0.921700039	0.729915
	Standard deviation	0.044807644	0.06069
Maximum extreme difference	Absolute	0.109195339	0.093113
	Correct	0.099193227	0.093113
	Negative	-0.109195339	-0.07782
Test statistics		0.109195339	0.093113
Asymptotic significance		.200[d]	.200[d]
Monte Carlo significance		0.2643	0.5068
99% confidence interval	Lower limit	0.252941629	0.493922
	Upper limit	0.275658371	0.519678

Table S5. Contribution of environmental variables and permutation importance

Environmental variable	Percent contribution	Permutation importance
bio18	34	55.6
bio14	33.9	3.3
alt	15.9	2
bio12	8	27.8
bio19	4.9	2.9
bio9	2	2.7
bio15	1.2	5.8
bio2	0.1	0

TableS S6. Centroid coordinates of distribution and migration distance

Period	Longitude	Latitude	Migration Distance (km）
Current	101.894	36.8427	0
Last Inter Glacial	100.714	37.7299	114.0327
Mid Holocene	101.428	36.876	41.6242
SSP126 (2041-2060)	101.991	36.7313	15.1016
SSP370 (2041-2061)	101.906	36.8111	11.6643

SSP585 (2041-2062)	101.834	36.8537	7.9687
SSP126 (2081-2100)	101.983	36.7535	12.6955
SSP370 (2081-2101)	102.073	36.7444	8.0823
SSP585 (2081-2102)	102.307	36.5746	28.1455

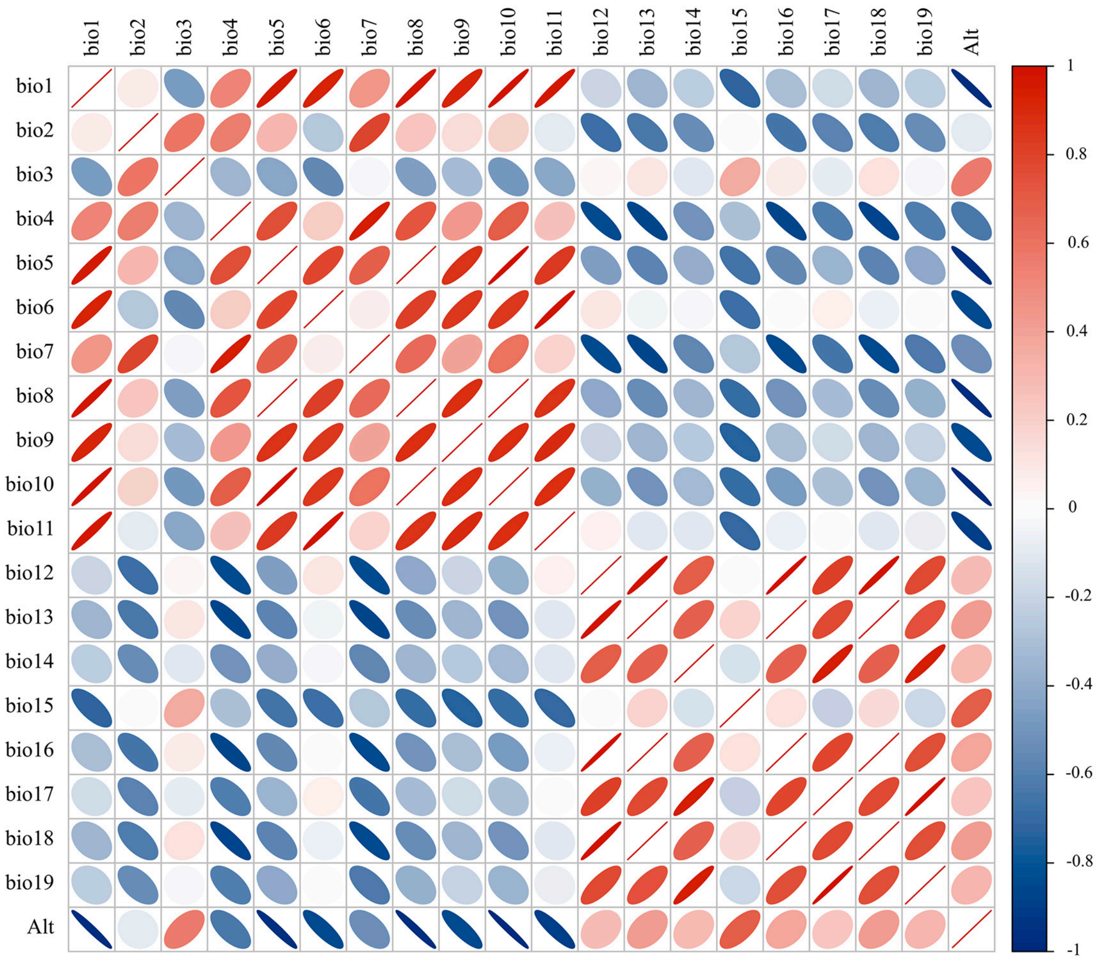

Figure S1. Correlation heatmap of environmental variables.

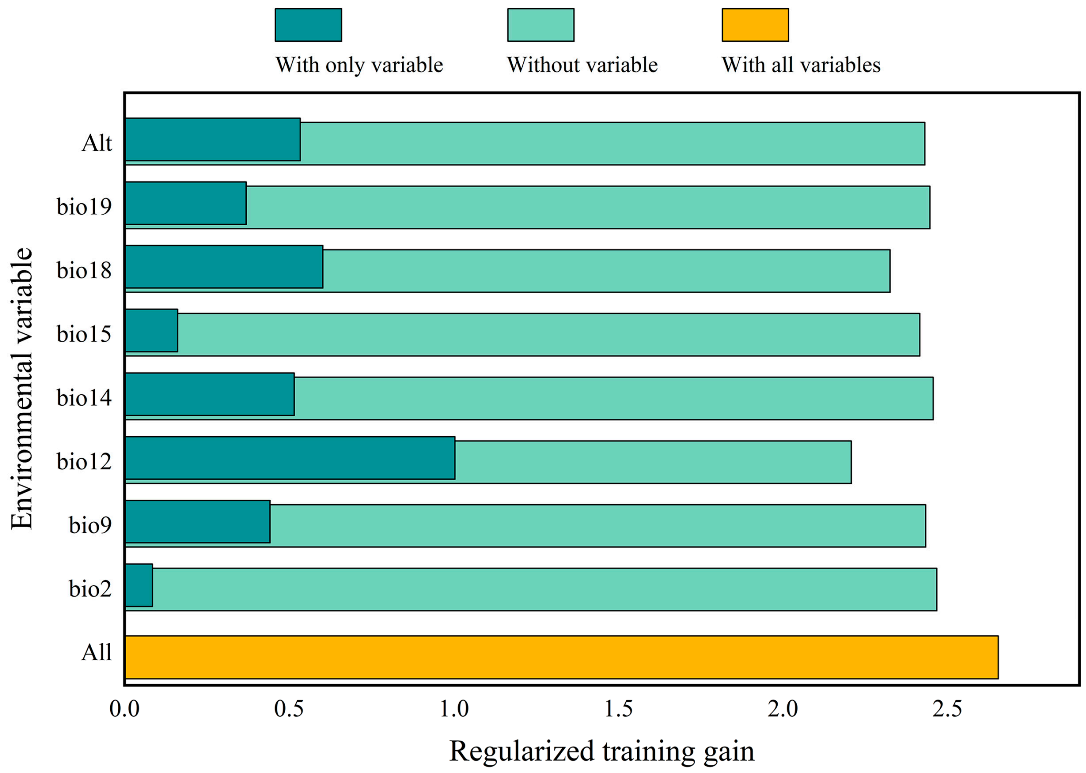

Figure S2. Results of the Jackknife test for primary environmental factors.
